# Ultrasonography of cutaneous nodular pseudolymphoma at 18 and 71 MHz

**DOI:** 10.1111/srt.13099

**Published:** 2021-08-29

**Authors:** Ximena Wortsman, Camila Ferreira‐Wortsman, Kharla Pizarro, Claudia Morales

**Affiliations:** ^1^ Institute for Diagnostic Imaging and Research of the Skin and Soft Tissues Santiago Chile; ^2^ Department of Dermatology Faculty of Medicine Universidad de Chile Santiago Chile; ^3^ Faculty of Medicine Universidad Finis Terrae Santiago Chile; ^4^ Department of Pathology Hospital San José Santiago Chile; ^5^ Department of Pathology Faculty of Medicine, Hospital Clínico Universidad de Chile Universidad de Chile Santiago Chile

**Keywords:** dermatologic ultrasound, skin ultrasound, lymphocytoma cutis, lymphocytoma cutis ultrasound, lymphoma ultrasound, Pseudolymphoma, pseudolymphoma ultrasound

## Abstract

Cutaneous pseudolymphomas are reactive lymphoproliferations. The most frequent type is nodular pseudolymphoma, and to date, their ultrasonographic appearance has not been reported. We reviewed the ultrasound images of histologically confirmed nodular types of pseudolymphomas studied with 18 and 71 MHz linear probes. All lesions were predominantly hypoechoic and presented prominent vascularity. Seventy percent of cases involved dermis and hypodermis, and 30% were only dermal. Seventy percent of cases showed internal hypoechoic globules, and 100% presented a teardrop sign, more clearly detected at 71 MHz. Ultrasound can support the diagnosis, assessment of the extent, and degree of vascularity of cutaneous nodular pseudolymphomas.

Dear Editor,

Cutaneous pseudolymphoma, also called lymphocytoma cutis and cutaneous lymphoid hyperplasia, is a heterogeneous group of polyclonal reactive lymphoproliferations that can clinically imitate cutaneous lymphomas and other dermatologic lesions.^1^ The pseudolymphomas present variable etiology, pathogenesis, clinical presentation, histology, and behavior.^1^, ^2^, ^3^, ^4^

On immunohistochemistry, they can be classified according to their predominant lymphocytic component in cutaneous B‐cell, T‐cell, or mixed pseudolymphomas.^1^, ^4^, ^5^ However, as shown in the literature, the histological diagnosis is usually made on hematoxylin‐eosin, being immunohistochemistry performed in cases suspicious of lymphomas.^1^, ^4^

Among the reported causes of cutaneous pseudolymphomas are infectious diseases, such as bacterias (e.g., Borrelia sp. and Treponema pallidum), viruses (e.g., Herpes virus sp., Molluscipoxvirus, and HIV), parasites (e.g., scabies and leishmania), drugs (e.g., anticonvulsants, antipsychotics, antihypertensives, antiarrhythmics, antibiotics, antirheumatics, anxiolytics, and Non‐steroidal anti‐inflammatory drugs (NSAIDs)), insect bites, and tattoos.^1^, ^4^, ^5^, ^6^

The nodular variant is the most common type of cutaneous pseudolymphomas. Clinically, it appears as a single erythematous nodule, commonly located in the head and neck; however, multiple nodules and other corporal locations are also described in the literature.^1^, ^2^, ^3^, ^4^, ^5^, ^6^, ^7^

To date, there are no reports in the literature on the ultrasound morphology of pseudolymphomas. Thus, this case series aimed to review the ultrasonographic appearance of the nodular type of pseudolymphomas.

The Institutional Review Board (Universidad de Chile) waived the need for consent for this retrospective study, but according to our internal protocol, all patients signed informed consent to publish their images. We reviewed the ultrasonographic images of cutaneous nodular pseudolymphomas cases of the period January 2018–December 2020.

The *inclusion criteria* were:

– Dermatologic evaluation performed previous to the ultrasound examination.
– Cases studied with color Doppler ultrasound devices working with linear probes presenting a maximum frequency of 18 and 71 MHz the same day.
– Histologic confirmation of diagnosis.

The *exclusion criteria* were:

– History of surgery, biopsy, or cosmetic procedures in the same or adjacent corporal regions previous to the ultrasound examination.
– Concomitant systemic or regional cutaneous disease.

All cases were studied first with a Logic E9 XD Clear ultrasound device (Logiq E 9 XD, General Electric Health System Clear, Waukesha, WI), using a compact linear probe with a frequency that ranges from 8 to 18 MHz; then, an ultrasound study was performed with a Vevo MD device using a linear probe that ranges from 29 to 71 MHz (UHF 70, Vevo MD, VisualSonics, Toronto, Ontario, Canada).

The protocol followed the guidelines for performing dermatologic ultrasound examinations, already published in the literature.^8^, ^9^

In all cases, the descriptive data, grayscale, and color Doppler ultrasound information, including spectral curve analysis, were recorded. The ultrasound data included: corporal location, skin layers involved, echogenicity, shape, epidermal upward displacement, diameters in all axes, and vascularity.

The vascularity was categorized as present or absent; the vessels' maximum thickness (mm) and the maximum peak systolic velocity (cm/s) of the arterial vessels within the lesions were registered in all cases.

Two ultrasonographic signs were investigated: the globules sign (i.e., hypoechoic nodular or pseudonodular areas within the lesions) and the teardrop sign (i.e., a hypoechoic structure resembling a teardrop or presenting a mainly triangular shape within the lesion).

Ten patients (mean age: 42 years; SD± 21; range: 6–63 years) met the criteria; of these, 70% were females, and the corporal distribution of the lesions was: 80% in the face and neck, 10% in the thoracic region, and 10% in the arm. Regarding the cutaneous layers involvement: 70% of cases were dermal and hypodermal (mostly upper hypodermal), and 30% only dermal.

The lesions' shape was slightly fusiform in 70% of lesions and mostly oval in 30%. All lesions were predominantly hypoechoic compared to the adjacent normal dermis, and 90% of cases showed an upward epidermal displacement.

Despite the presence or absence of hypodermal involvement, all cases demonstrated an increased echogenicity of the adjacent hypodermis.

Seventy percent of cases showed the globules sign, and 100% presented a teardrop sign, more clearly detected at 71 MHz and more prominent in the deep part of the lesions (Figures 1, 2, 3 and Figures S1–3).

**FIGURE 1:**
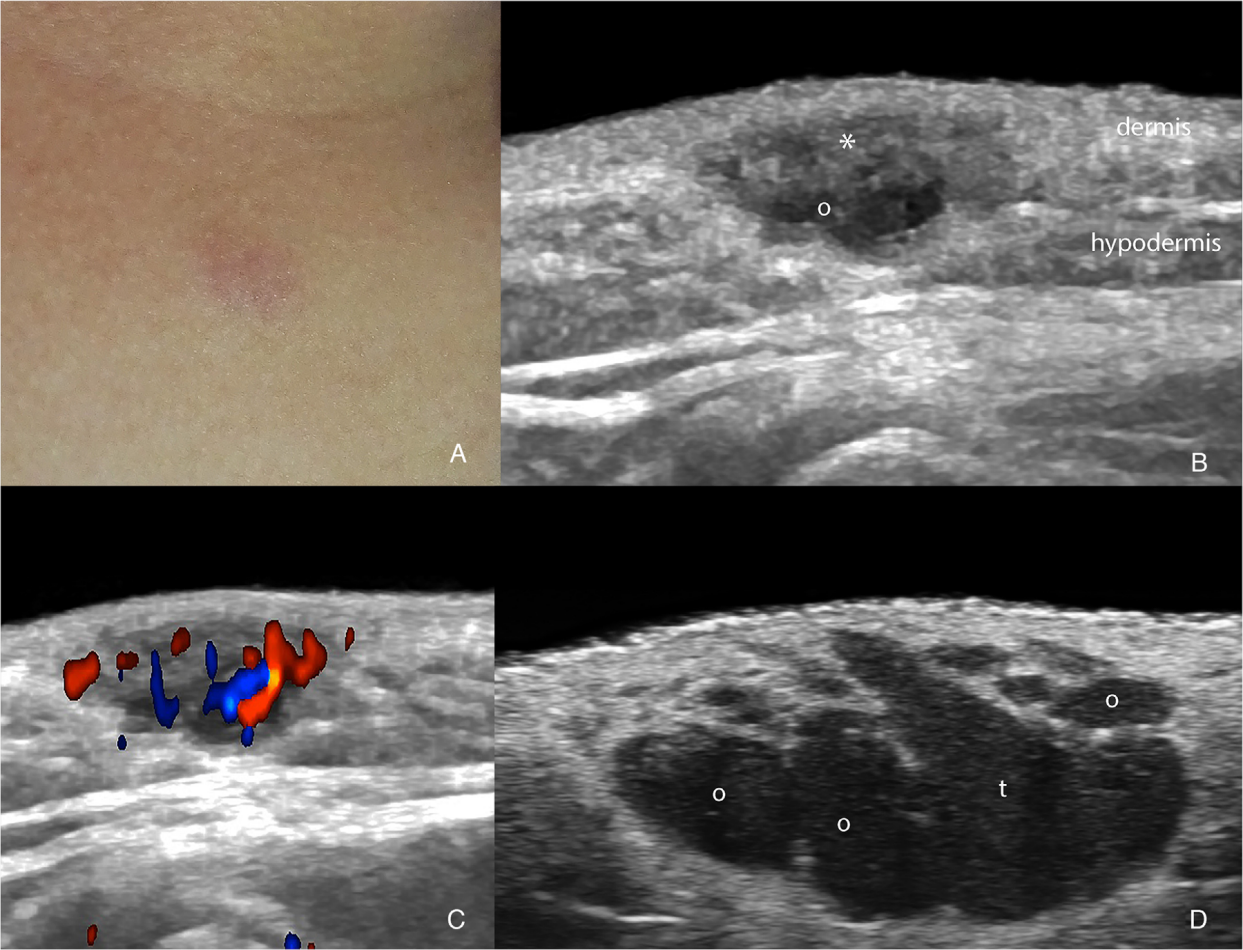
**Nodular pseudolymphoma**. Clinical‐ultrasonographic correlation. (A) Clinical image (6‐year‐old female; presternal region). (B–D) Ultrasonographic images. B and C at 18 MHz (B. grayscale and C. color Doppler) and (D) grayscale at 71 MHz demonstrate a slightly fusiform hypoechoic dermal and hypodermal structure (*) with a softly rising of the epidermis. On color Doppler, there is hypervascularity within the lesion. At 71 MHz, it is possible to better define the globules (o) and teardrop (t) signs at the lesion's bottom part

**FIGURE 2:**
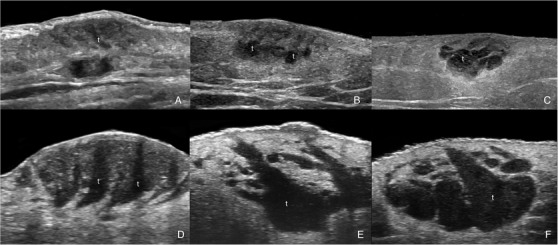
**Nodular pseudolymphomas teardrop signs (t) in different cases**. A–C at 18 MHz and D–F at 71 MHz

**FIGURE 3:**
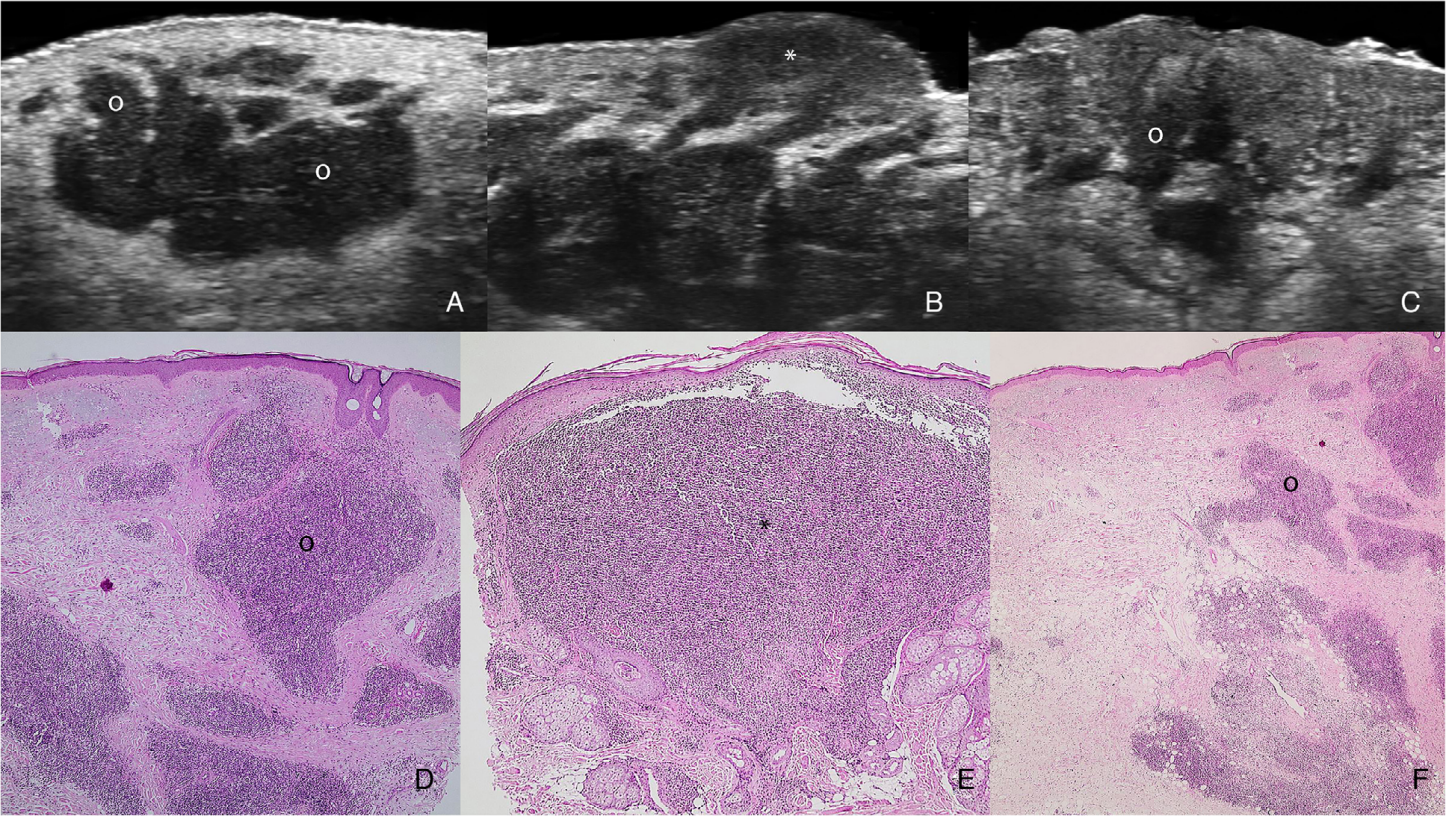
**Ultrasonographic histologic correlation**. (A–C) Grayscale images at 71 MHz demonstrate multiple hypoechoic globules (o) within the lesional areas (*). (D–F) Histology (hematoxylin‐eosin, 4x magnification) shows the lesional areas (*) with compact clusters of lymphocytes and inflammatory infiltrates (o), mostly polymorphic cells, that resemble the globules' ultrasonographic structures

The mean diameters of the lesions were transverse: 1.0 cm ± 0.3, thickness: 0.4 cm ± 0.2, and longitudinal: 0.9 cm ± 0.4. No signs of calcium deposits were detected within or in the periphery of the lesions.

All cases presented prominent internal vascularity. The lesional vessels' mean thickness was: 0.8 mm ± 0.2, and the maximum peak systolic velocity of the arterial vessels within the lesions was 10.7 cm/s ± 5.6).

Nodular cutaneous pseudolymphomas may be simulators of other dermatologic lesions, including malignant lesions, such as lymphomas.^4^, ^5^, ^7^ The provision of ultrasound patterns of pseudolymphomas may contribute to distinguishing this condition better; however, ultrasound is not a substitute for pathology.

The common presence of the teardrop and the globules signs within the lesions can orient us to the diagnosis of pseudolymphomas. Hypoechoic nodules and pseudonodules are also reported in cutaneous lymphomas;^10^ however, they are described as ill‐defined, which would differ from the mostly well‐defined borders of the globules and teardrop‐shaped hypoechoic structures. Nevertheless, further investigation, including a larger number of cases, is needed to confirm that. The globules and the teardrop signs correlate well on histology with compact clusters of lymphocytes. In the teardrop sign, the hypoechoic structures seem to follow the hair follicles' axis; however, this would be difficult to confirm on pathology due to the histologic slices' different axes.

A slightly fusiform shape with a predominant superficial involvement (dermis and upper hypodermis) and upward displacement of the epidermis can also support the diagnosis of pseudolymphomas over lymphomas. Moreover, superficial ultrasonographic alterations in the dermis and upper hypodermis in nodular pseudolymphomas correlate well with the histologic descriptions.^1^, ^3^, ^4^, ^5^ On pathology, one of the signs that favor the diagnosis of pseudolymphomas over lymphomas is the presence of dermal inflammatory infiltrates. Conversely, lymphomas tend to involve the hypodermis, including the deep subcutis, more easily.^5^

The hypervascularity found on nodular pseudolymphomas is probably due to their inflammatory nature that gathers lymphocytes, eosinophils, plasma cells, and histiocytes.^4^, ^5^, ^6^

The study with 71 MHz provided a high axial spatial resolution that reaches 30 microns^11^ and generates a more detailed zoom on the lesions' echostructure that could better discriminate the globules and teardrop signs.

Besides supporting the diagnosis, ultrasonography allows assessing the extent and degree of vascularity of the lesions in all cases. This would help the management of these lesions with more objective information and potentially monitor the lesions.

Among the study's limitations are the low number of cases and the devices' axial spatial resolution (100 microns at 18 MHz and 30 microns at 71 MHz).^11^ Nevertheless, the details of these images would not be possible to detect on Computed Tomography (CT) or Magnetic Resonance Imagings (MRIs) that present lower axial spatial resolutions.

In conclusion, ultrasound can support the diagnosis of cutaneous nodular pseudolymphomas. Further research is needed on this topic.

## Supporting information


**Figure S1**. Nodular pseudolymphoma. Clinical‐ultrasonographic correlation. (A) Clinical photograph of the lesion (50‐year‐old female, left cheek). (B–D) Ultrasound. B and C at 18 MHz (B, grayscale and C, color Doppler), and (D) Grayscale at 71 MHz demonstrate oval‐shaped hypoechoic dermal and upper hypodermal structure. Notice upward epidermal displacement, the hypoechoic globules (o), and the teardrop (t) signs. On color Doppler, there is prominent hypervascularity within the lesion.

**Figure S2**. Nodular pseudolymphoma. Clinical‐ultrasonographic correlation. (A) Clinical photograph (12‐year‐old female; submental region). (B) Dermoscopy view of the lesion. (C) Color Doppler ultrasound at 18 MHz demonstrates a slightly fusiform, hypoechoic dermal, and hypodermal lesion (*) with an upward displacement of the epidermis and internal hypervascularity. (D) Grayscale at 71 MHz, it is possible to detect hypoechoic globules (o) and teardrop (t) signs at the bottom of the lesion (*).

Figure S3. Nodular pseudolymphomas globules signs (o) in different cases. A–C at 18 MHz and D–F at 71 MHz.
