# Supplementary figures and images for: Ultrasonography of cutaneous nodular pseudolymphoma at 18 and 71 MHz
Source: Skin Res Technol. 2021 Aug 29;28(1):176–9. doi: 10.1111/srt.13099 (PMC9907700; doi:10.1111/srt.13099)

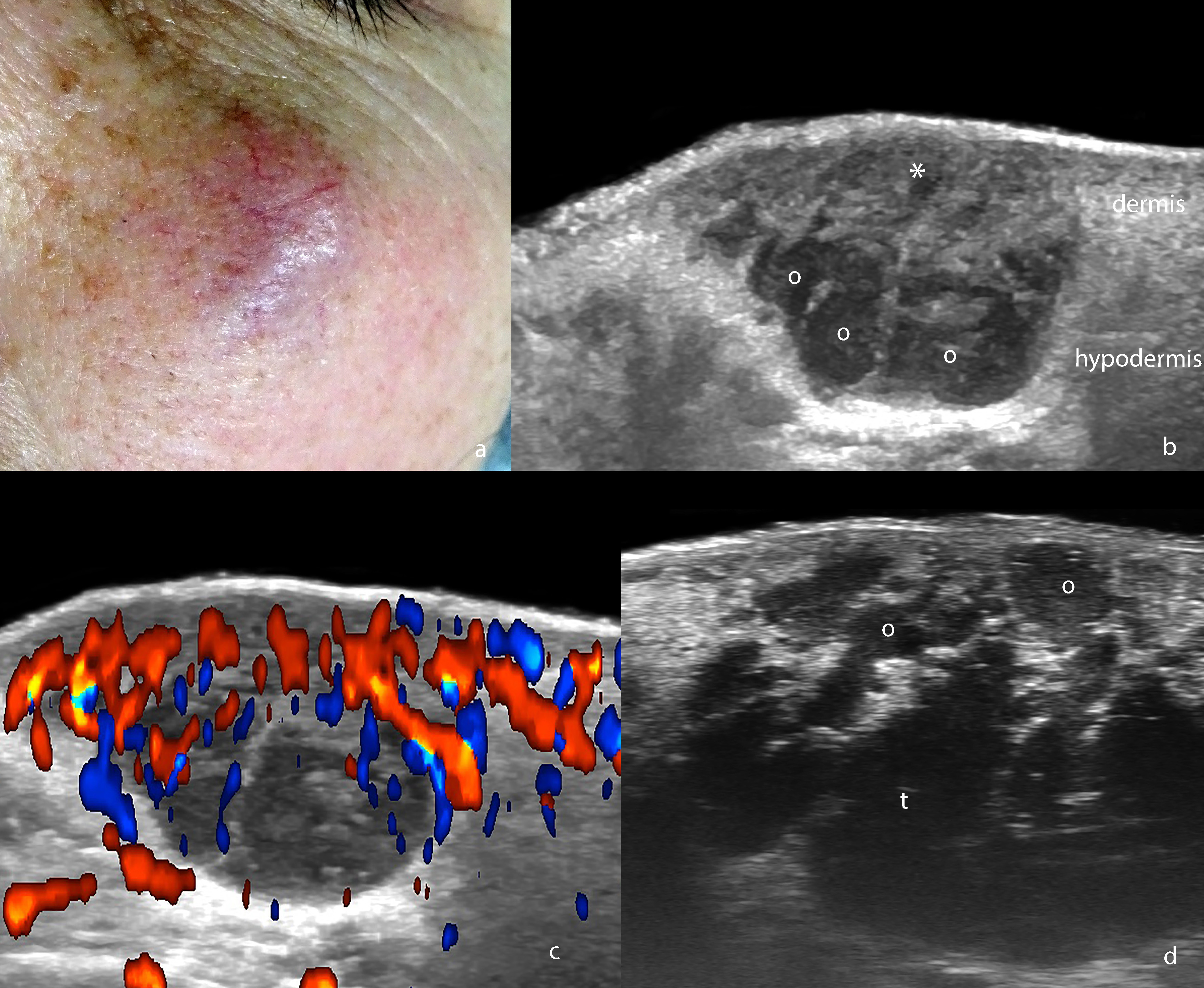

Supplement: Supplementary file 1 — Figure S1. Nodular pseudolymphoma. Clinical‐ultrasonographic correlation. (A) Clinical photograph of the lesion (50‐year‐old female, left cheek). (B–D) Ultrasound. B and C at 18 MHz (B, grayscale and C, color Doppler), and (D) Grayscale at 71 MHz demonstrate oval‐shaped hypoechoic dermal and upper hypodermal structure. Notice upward epidermal displacement, the hypoechoic globules (o), and the teardrop (t) signs. On color Doppler, there is prominent hypervascularity within the lesion. [file SRT-28-176-s001.jpg]

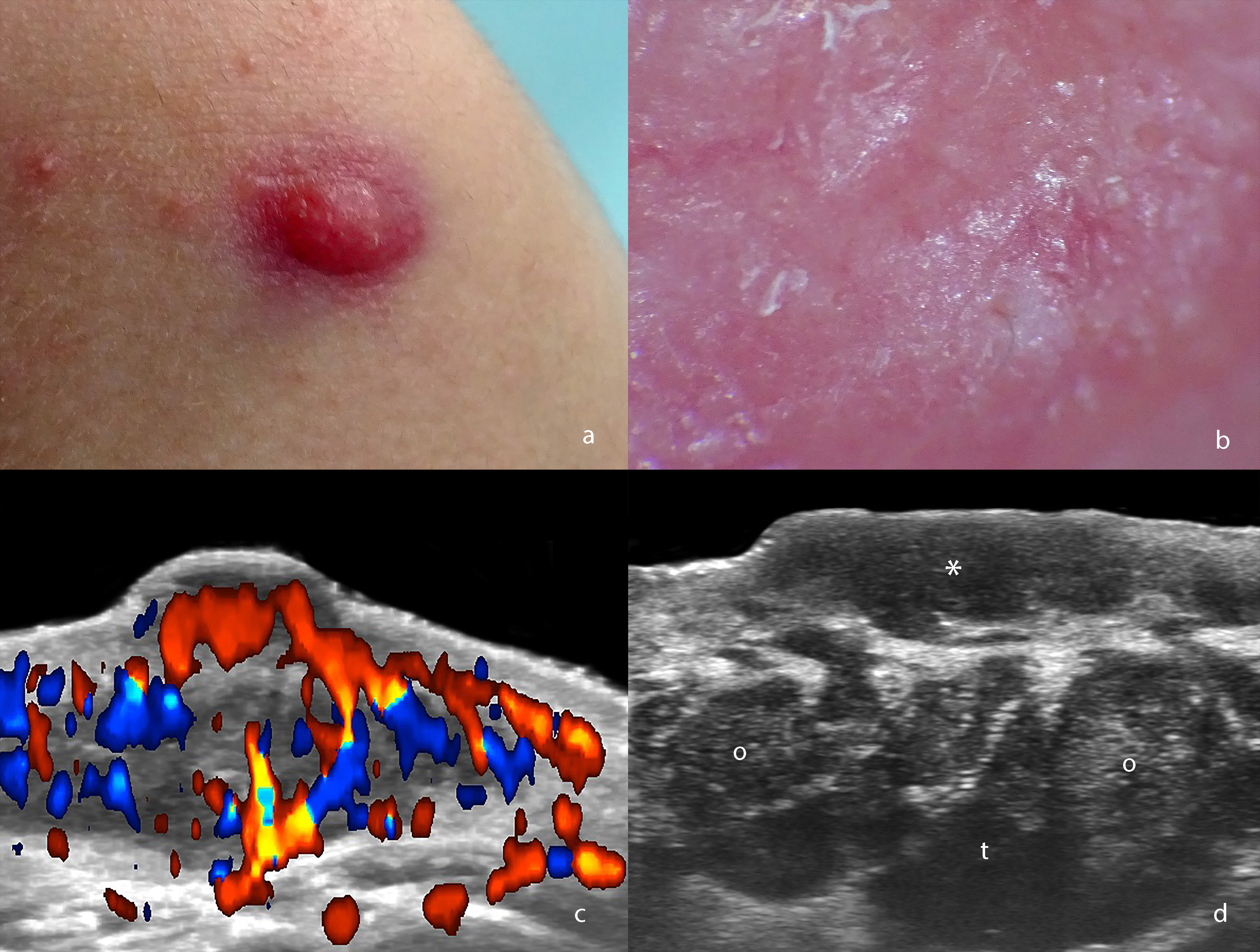

Supplement: Supplementary file 2 — Figure S2. Nodular pseudolymphoma. Clinical‐ultrasonographic correlation. (A) Clinical photograph (12‐year‐old female; submental region). (B) Dermoscopy view of the lesion. (C) Color Doppler ultrasound at 18 MHz demonstrates a slightly fusiform, hypoechoic dermal, and hypodermal lesion (*) with an upward displacement of the epidermis and internal hypervascularity. (D) Grayscale at 71 MHz, it is possible to detect hypoechoic globules (o) and teardrop (t) signs at the bottom of the lesion (*). [file SRT-28-176-s002.jpg]

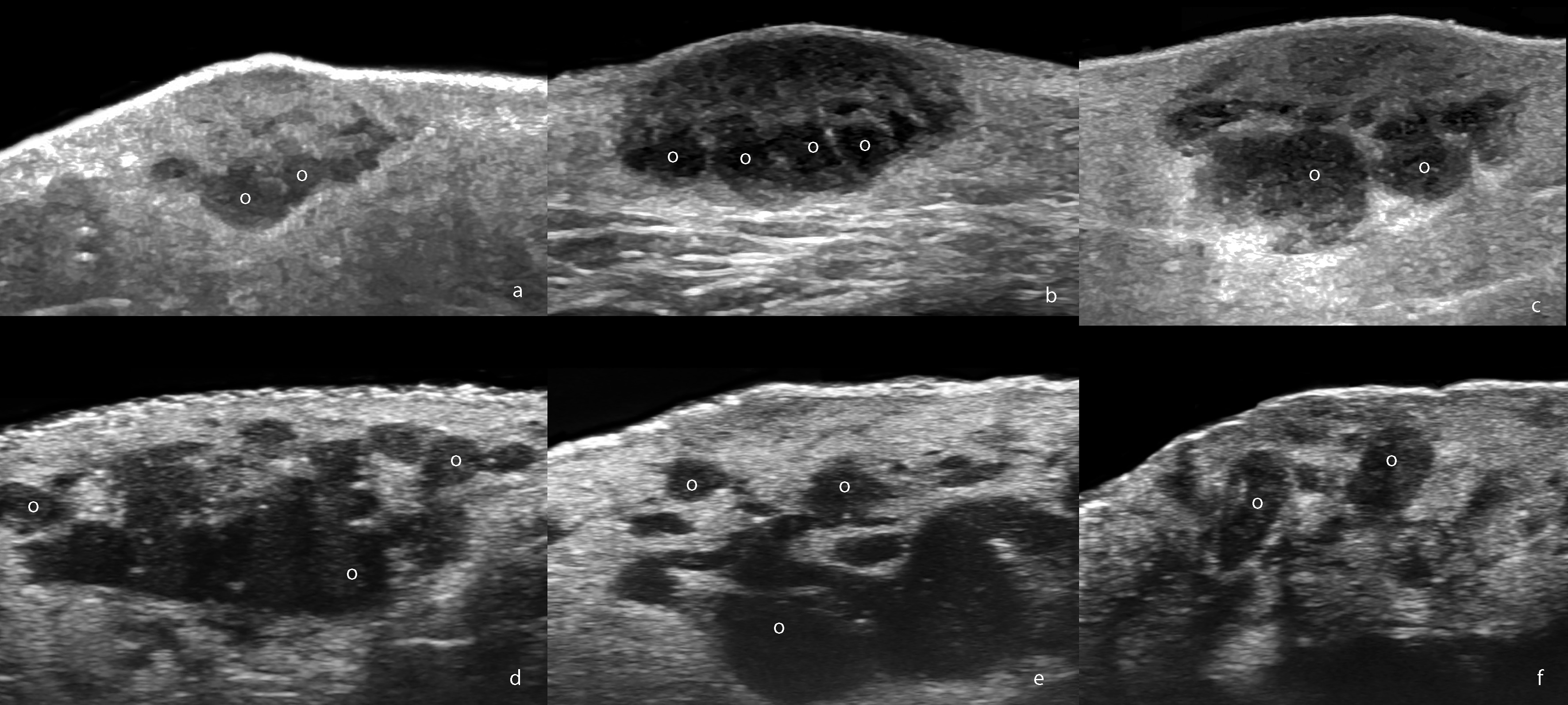

Supplement: Supplementary file 3 — Figure S3. Nodular pseudolymphomas globules signs (o) in different cases. A–C at 18 MHz and D–F at 71 MHz. [file SRT-28-176-s003.jpg]
